# Emerging Precision Therapeutic Strategies in Pancreatic Ductal Adenocarcinoma: KRAS Targeting, DDR Vulnerabilities, Immune Redirection, and Tumor Microenvironment Remodeling

**DOI:** 10.3390/pharmaceutics18080934

**Published:** 2026-07-29

**Authors:** Jun Kim, Seounghun Kang

**Affiliations:** 1 Department of Chemistry, Soongsil University, Seoul 06978, Republic of Korea; seallingfx@gmail.com; 2 AI-Bio Convergence Research Institute, Soongsil University, Seoul 06978, Republic of Korea

**Keywords:** pancreatic ductal adenocarcinoma, KRAS inhibitor, DNA damage response, immune redirection, tumor microenvironment, precision oncology

## Abstract

**Background/Objectives**: Pancreatic ductal adenocarcinoma (PDAC) remains one of the most lethal malignancies because of aggressive tumor biology, pervasive therapeutic resistance, rapid adaptive reprogramming, and a profoundly immunosuppressive tumor microenvironment. This review summarizes recent advances in KRAS-targeted therapies, DNA damage response (DDR)-directed strategies, immune-redirection platforms, and tumor microenvironment modulation. **Methods**: We conducted a narrative review of peer-reviewed publications, clinical trial reports, trial registries, and selected conference data addressing emerging therapeutic strategies for PDAC. **Results**: KRAS-targeted therapies, including KRAS G12D-selective and multi-selective RAS(ON) inhibitors, have demonstrated substantial preclinical and emerging clinical activity while revealing diverse mechanisms of adaptive resistance. DDR-directed approaches are expanding beyond BRCA-mutated disease toward functional homologous recombination deficiency, replication stress, and synthetic lethality. In parallel, bispecific antibodies, T-cell engagers, and CAR-T-cell therapies have generated preliminary evidence of antitumor activity, although stromal exclusion, antigen heterogeneity, and immune suppression remain major barriers. These advances indicate that therapeutic resistance in PDAC is a dynamic process involving interconnected oncogenic, genomic, stromal, metabolic, and immune mechanisms. **Conclusions**: Future progress will require biologically informed treatment frameworks integrating complementary therapeutic modalities according to baseline tumor biology and treatment-induced adaptive states, supported by longitudinal biomarkers and rational clinical trial design.

## 1. Introduction

Pancreatic ductal adenocarcinoma (PDAC) remains one of the most lethal solid malignancies worldwide, with a 5-year survival rate that remains below 15% despite decades of therapeutic advances [1]. The aggressive clinical course of PDAC is largely attributable to late-stage diagnosis, rapid metastatic dissemination, profound therapeutic resistance, and high postoperative recurrence rates [2,3]. Even among patients who undergo curative-intent resection, the majority eventually experience disease recurrence, highlighting the persistent limitations of current treatment strategies [4].

Over the past decade, combination cytotoxic chemotherapy regimens such as FOLFIRINOX (oxaliplatin, irinotecan, leucovorin, and fluorouracil) and gemcitabine plus nab-paclitaxel (GnP) have modestly improved survival outcomes in metastatic PDAC and remain the current standard-of-care in most clinical settings [5,6]. Nevertheless, the overall clinical benefit remains limited by substantial systemic toxicity, the short-lived durability of response, and the rapid emergence of acquired resistance [3]. Moreover, unlike several other solid tumors in which molecularly targeted therapies and immune checkpoint inhibitors have dramatically transformed treatment paradigms, PDAC has historically demonstrated limited responsiveness to both precision oncology and immunotherapeutic approaches [7].

The therapeutic refractoriness of PDAC is driven by a uniquely complex and highly adaptive tumor biology. Oncogenic KRAS mutations are present in more than 90% of PDAC cases and function as a central driver of tumor initiation, metabolic reprogramming, and survival signaling [8]. Beyond KRAS dependency, PDAC is characterized by a dense desmoplastic stroma, extensive cancer-associated fibroblast (CAF) accumulation, hypovascularity, and a profoundly immunosuppressive tumor microenvironment (TME), collectively generating both physical and biological barriers to effective therapy [9,10]. In addition, substantial intratumoral heterogeneity and adaptive signaling rewiring further contribute to rapid therapeutic resistance against both cytotoxic chemotherapy and molecularly targeted therapies in PDAC [3].

Recent advances in translational oncology, however, have begun to reshape the therapeutic landscape of PDAC. Historically regarded as one of the most therapeutically refractory malignancies, PDAC is increasingly becoming the focus of mechanism-driven therapeutic development. Emerging strategies include direct KRAS inhibition, DNA damage response (DDR)-targeted therapy, immune-redirection platforms such as T-cell engagers (TCEs) and bispecific antibodies, tumor microenvironment remodeling, and biomarker-guided precision medicine [11,12]. In addition to KRAS-directed therapies, several biomarker-guided treatment strategies have emerged for molecularly defined subsets of PDAC. Although individually rare, actionable alterations including NRG1 fusions, NTRK fusions, RET fusions, and BRAF V600E mutations have demonstrated clinically meaningful responses to matched targeted therapies, highlighting the growing role of comprehensive genomic profiling and precision oncology in PDAC management [13,14,15,16]. Despite their low prevalence, these molecular alterations collectively illustrate the gradual transition of PDAC toward a precision oncology paradigm. Importantly, these approaches are increasingly being integrated through rational combination strategies designed to overcome the multifactorial resistance mechanisms inherent to PDAC biology.

Among these developments, the emergence of KRAS-targeted agents represents a particularly significant milestone. Novel KRAS G12D inhibitors, pan-RAS inhibitors, and RAS(ON) inhibitors have demonstrated encouraging preclinical and early clinical activity, renewing optimism for direct targeting of KRAS-driven signaling networks in PDAC [17,18]. Simultaneously, DDR-directed therapeutic strategies are expanding beyond germline BRCA-mutated populations toward broader concepts of homologous recombination deficiency (HRD), replication stress vulnerability, and synthetic lethality [19]. In parallel, immunotherapeutic development in PDAC is shifting away from immune checkpoint blockade alone toward strategies focused on immune redirection and tumor microenvironment reprogramming, including CD40 agonists, cancer vaccines, bispecific antibodies, and CLDN18.2-targeted platforms [7,20].

Importantly, accumulating evidence suggests that therapeutic failure in PDAC rarely results from a single dominant resistance mechanism. Rather, PDAC behaves as a highly adaptive network disease characterized by simultaneous oncogenic redundancy, stromal protection, metabolic plasticity, and immune exclusion. Consequently, durable therapeutic responses will likely require multidimensional combination strategies capable of simultaneously disrupting interconnected resistance networks rather than targeting isolated molecular pathways alone.

**Figure 1:**
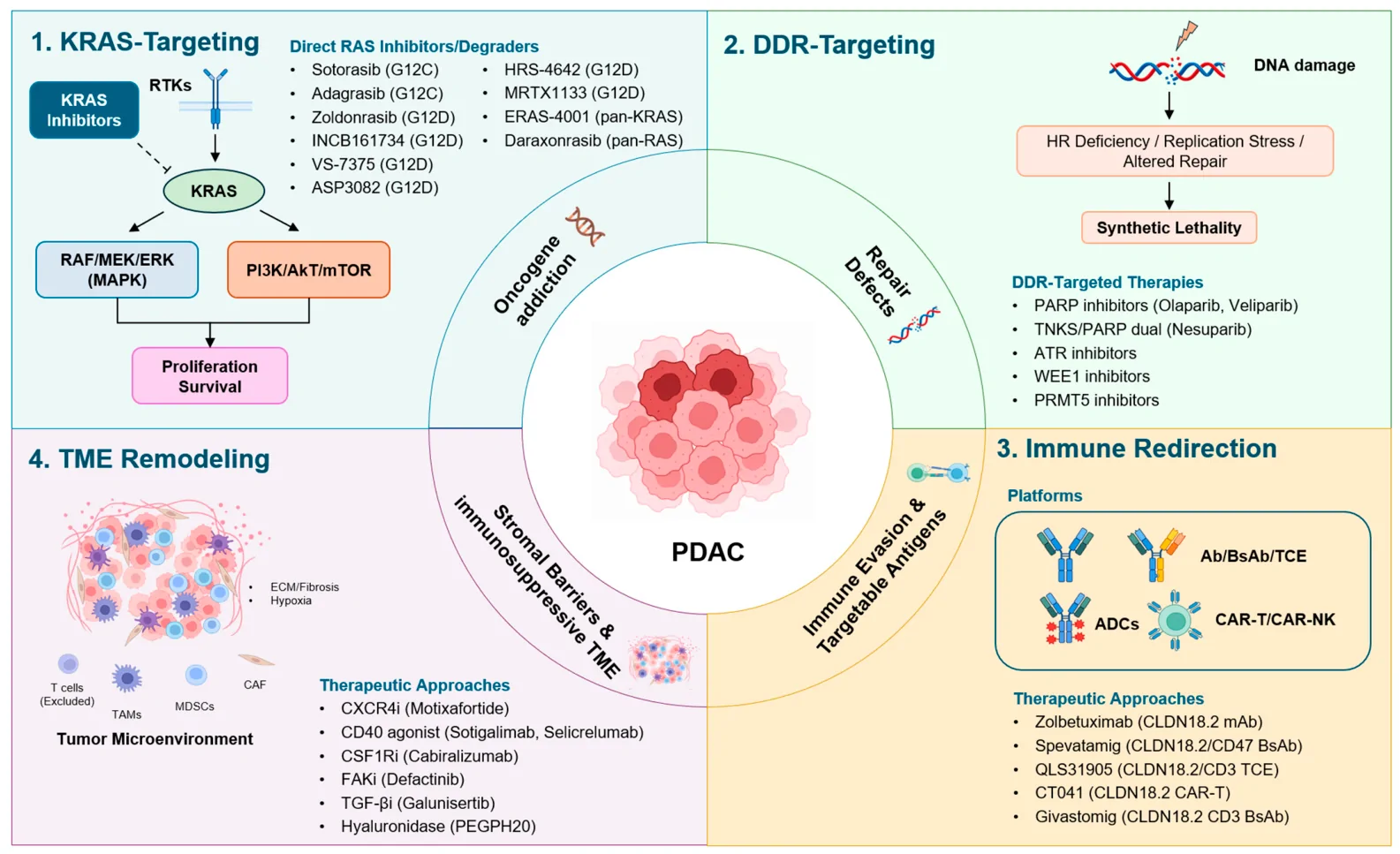
Emerging therapeutic paradigms and mechanism-based combination strategies in pancreatic ductal adenocarcinoma (PDAC). The schematic summarizes four major therapeutic domains: KRAS targeting, DNA damage response (DDR)-targeted therapy, immune redirection, and tumor microenvironment (TME) remodeling. KRAS-targeting strategies include direct inhibitors and rational combination approaches targeting receptor tyrosine kinase (RTK), SHP2, phosphoinositide 3-kinase (PI3K), and mitogen-activated protein kinase (MAPK) signaling. DDR-targeted therapies exploit homologous recombination (HR) deficiency, replication stress, and synthetic lethality through poly(ADP-ribose) polymerase (PARP), dual tankyrase (TNKS)/PARP, ataxia telangiectasia and Rad3-related (ATR), WEE1, and protein arginine methyltransferase 5 (PRMT5) inhibitors. Immune-redirection approaches include monoclonal antibodies (mAbs), bispecific antibodies (BsAbs), T-cell engagers (TCEs), antibody–drug conjugates (ADCs), chimeric antigen receptor T-cell (CAR-T), and CAR-natural killer (CAR-NK) therapies targeting tumor-associated antigens such as claudin 18.2 (CLDN18.2), mesothelin, carcinoembryonic antigen (CEA), epidermal growth factor receptor/human epidermal growth factor receptor 2 (EGFR/HER2), and mucin 1 (MUC1). TME remodeling strategies include C-X-C chemokine receptor type 4 (CXCR4), CD40, colony-stimulating factor 1 receptor (CSF1R), focal adhesion kinase (FAK), and transforming growth factor-beta (TGF-β) inhibitors, together with hyaluronidase-based stromal depletion. Collectively, these therapeutic domains illustrate the shift from conventional chemotherapy toward biomarker-driven precision and rational combination strategies in PDAC.

In this review, we summarize the evolving clinical development landscape of PDAC from a mechanistic and translational perspective, with particular emphasis on KRAS-targeted therapies, DDR-directed strategies, immune-redirection platforms, and tumor microenvironment remodeling, as shown in Figure 1. We further discuss emerging biomarkers, rational combination strategies, and future directions toward precision-combination therapeutic approaches in PDAC.

## 2. Literature Search Strategy

This narrative review was based on searches of PubMed, Web of Science, Scopus, and ClinicalTrials.gov, with the final search performed on 17 July 2026. Studies published between January 2019 and July 2026 were preferentially included, while earlier landmark studies were retained when necessary to provide historical or mechanistic context. Search terms included “pancreatic ductal adenocarcinoma,” “KRAS inhibitor,” “RAS(ON),” “DNA damage response,” “PARP inhibitor,” “ATR inhibitor,” “WEE1 inhibitor,” “PRMT5,” “MTAP deletion,” “tumor metabolism,” “autophagy,” “cancer vaccine,” “tumor microenvironment,” “CLDN18.2,” “T-cell engager,” “bispecific antibody,” “CAR-T,” “CD40,” “CXCR4,” “FAK,” and “TGF-β.” Priority was given to peer-reviewed clinical and translational studies. Conference abstracts and clinical trial registries were considered when peer-reviewed evidence was unavailable for rapidly emerging agents and are identified as preliminary where appropriate. Both positive and negative clinical studies were included to reduce selective reporting bias. Because this was a narrative rather than systematic review, no formal meta-analysis or risk-of-bias assessment was performed. Landmark studies published before 2019 were additionally cited when necessary to provide historical context.

## 3. Current Therapeutic Landscape and Biological Barriers in PDAC

### 3.1. Current Standard-of-Care Therapies

Despite ongoing advances in precision oncology, systemic treatment for PDAC remains largely dependent on cytotoxic chemotherapy. In the metastatic setting, two multi-agent regimens currently constitute the backbone of first-line therapy: FOLFIRINOX and GnP (Table 1).

The PRODIGE 4/ACCORD 11 trial established FOLFIRINOX as a standard first-line regimen for patients with good performance status, demonstrating significant survival benefits compared with gemcitabine monotherapy. Median overall survival (mOS) reached 11.1 months with FOLFIRINOX versus 6.8 months with gemcitabine, while median progression-free survival (mPFS) improved from 3.3 to 6.4 months [5]. However, the regimen was associated with considerable hematologic and gastrointestinal toxicity, limiting its use primarily to medically fit patients.

GnP later emerged as an alternative first-line option following the MPACT trial. In this study, GnP demonstrated superior survival compared with gemcitabine alone, with an mOS of 8.5 months versus 6.7 months and an mPFS of 5.5 months versus 3.7 months, respectively [6]. Although generally considered more tolerable and broadly applicable than FOLFIRINOX, cumulative neuropathy, treatment resistance, and limited durability of response remain major clinical challenges.

For patients with germline BRCA-mutated PDAC, maintenance therapy with the PARP inhibitor olaparib has introduced a biomarker-driven treatment strategy. In the phase III POLO trial, maintenance olaparib significantly prolonged mPFS compared with placebo (7.4 vs. 3.8 months) following platinum-based chemotherapy, although overall survival benefit was less definitive [19]. Nonetheless, only a relatively small subset of patients currently qualify for this approach.

More recently, efforts to optimize first-line cytotoxic chemotherapy have led to the development of the NALIRIFOX regimen (nanoliposomal irinotecan, fluorouracil, leucovorin, and oxaliplatin). The NAPOLI-3 trial recently evaluated nanoliposomal irinotecan combined with fluorouracil, leucovorin, and oxaliplatin (NALIRIFOX) as a first-line regimen for metastatic PDAC, demonstrating improved overall survival compared with GnP (11.1 vs. 9.2 months) [20]. The introduction of NALIRIFOX further reflects the ongoing effort to optimize cytotoxic backbone regimens in PDAC. However, despite incremental improvements in survival, most patients ultimately develop rapid therapeutic resistance, and durable disease control remains uncommon.

Collectively, current standard-of-care therapies in PDAC continue to provide only modest survival benefits, underscoring the urgent need for mechanism-driven therapeutic strategies capable of overcoming the profound biological resistance characteristic of this disease.

### 3.2. Mechanisms Underlying Therapeutic Refractoriness

The limited efficacy of current therapies in PDAC is driven by a highly resistant and adaptive tumor biology. One of the defining features of PDAC is its dense desmoplastic stroma, composed of extracellular matrix proteins, cancer-associated fibroblasts (CAFs), and immunosuppressive immune cells, which collectively impair vascular perfusion and intratumoral drug delivery [9,10]. Elevated interstitial pressure and hypovascularity further restrict effective penetration of cytotoxic agents and immune effector cells, thereby contributing to both therapeutic resistance and immune exclusion [21].

At the molecular level, constitutive KRAS activation sustains oncogenic signaling, metabolic rewiring, and resistance to apoptosis [8]. However, increasing evidence suggests that PDAC exhibits partial rather than absolute KRAS dependency. Inhibition of KRAS signaling frequently induces compensatory bypass activation through receptor tyrosine kinases (RTKs), SHP2 signaling, and alternative downstream survival pathways, enabling rapid restoration of oncogenic signaling output despite initial pathway suppression [11].

PDAC also exhibits a profoundly immunosuppressive tumor microenvironment characterized by regulatory T cells, tumor-associated macrophages, and myeloid-derived suppressor cells, resulting in poor T-cell infiltration and limited responsiveness to immune checkpoint blockade [7]. Importantly, these stromal, oncogenic, metabolic, and immune-mediated resistance mechanisms do not operate independently. Rather, PDAC behaves as an interconnected adaptive ecosystem in which stromal remodeling, metabolic plasticity, immune suppression, and signaling redundancy collectively limit the durability of both cytotoxic chemotherapy and precision-targeted therapies.

In addition, substantial intratumoral heterogeneity and phenotypic plasticity further accelerate therapeutic adaptation under selective treatment pressure [22]. Collectively, these findings suggest that effective treatment of PDAC will likely require multidimensional therapeutic strategies capable of simultaneously disrupting tumor-intrinsic oncogenic signaling, stromal-mediated protection, and immune-evasive microenvironmental networks.

### 3.3. Current Clinical Unmet Needs in PDAC

Despite modest advances in systemic therapy, PDAC remains associated with poor long-term outcomes and limited durable responses [3]. Although FOLFIRINOX and GnP have improved median survival, most patients ultimately develop rapid disease progression due to intrinsic or acquired resistance [5,6].

A major unmet need in PDAC is the limited availability of actionable molecular targets. Currently, only a small subset of patients benefit from biomarker-driven therapies such as PARP inhibitors for germline BRCA-mutated disease or immune checkpoint inhibitors for MSI-high tumors [19]. Furthermore, conventional immunotherapy has shown minimal efficacy in PDAC because of its immunologically “cold” microenvironment and extensive myeloid-mediated immune suppression [7].

Another important challenge is the lack of effective strategies capable of overcoming stromal barriers, adaptive signaling pathways, and tumor heterogeneity simultaneously. Increasing evidence suggests that successful treatment of PDAC will likely require rational combination approaches integrating KRAS inhibition, DDR targeting, immune redirection, and tumor microenvironment remodeling. Accordingly, the development of biomarker-guided precision-combination strategies remains a major priority in PDAC therapeutics.

## 4. KRAS-Targeted Therapeutic Development

### 4.1. KRAS Biology and Oncogenic Signaling in PDAC

KRAS mutations represent the central oncogenic driver in PDAC, occurring in more than 90% of cases and constituting one of the earliest genetic events during pancreatic tumorigenesis [8,23,24]. Among these alterations, KRAS G12D is the predominant subtype, followed by G12V and G12R mutations, whereas KRAS G12C mutations are relatively uncommon in PDAC compared with lung cancer [25]. Constitutive KRAS activation promotes persistent downstream signaling through multiple effector pathways, including RAF–MEK–ERK, PI3K–AKT–mTOR, and RalGDS signaling, thereby driving tumor proliferation, survival, invasion, and metastatic progression [26].

Beyond its canonical role in tumor growth, oncogenic KRAS profoundly reshapes the metabolic and microenvironmental landscape of PDAC. KRAS-driven metabolic rewiring enhances glucose uptake, glycolysis, glutamine utilization, autophagy, and redox homeostasis, enabling tumor cells to survive under hypoxic and nutrient-deprived conditions characteristic of the PDAC microenvironment [27]. KRAS signaling also contributes to stromal remodeling and immune suppression through cytokine secretion, fibroblast activation, and recruitment of myeloid-derived suppressor cells and tumor-associated macrophages [28].

Importantly, KRAS signaling in PDAC extends beyond a simple proliferative oncogenic driver. Chronic KRAS activation orchestrates a broader adaptive tumor ecosystem involving metabolic plasticity, stromal remodeling, immune suppression, and replication stress tolerance. Consequently, therapeutic targeting of KRAS in PDAC may require simultaneous disruption of these KRAS-dependent adaptive networks rather than inhibition of oncogenic signaling alone.

Increasing evidence further suggests that PDAC exhibits partial rather than absolute KRAS dependency. Persistent KRAS signaling facilitates adaptive resistance through extensive crosstalk with RTKs, SHP2-mediated signaling, and compensatory downstream survival pathways [11]. Moreover, distinct KRAS mutation subtypes may exhibit biologically heterogeneous behavior, potentially influencing therapeutic sensitivity, metabolic dependency, and clinical outcomes [29].

Collectively, these findings establish KRAS not only as a dominant oncogenic driver in PDAC, but also as a central regulator of tumor metabolism, stromal interaction, immune evasion, and therapeutic adaptation. This biological complexity may partially explain why durable clinical responses to KRAS inhibition remain challenging despite substantial advances in KRAS-targeted drug development.

### 4.2. Direct KRAS Inhibition Strategies

For decades, KRAS was considered an “undruggable” target because of its high affinity for GTP/GDP and the absence of suitable small-molecule binding pockets [26]. However, recent advances in structural biology and medicinal chemistry have fundamentally reshaped the therapeutic landscape of KRAS-mutant cancers, leading to the rapid development of allele-specific, pan-RAS, and RAS(ON)-selective inhibitors (Table 2).

Initial clinical proof-of-concept was achieved with KRAS G12C inhibitors such as sotorasib and adagrasib. In the phase I/II CodeBreaK100 trial, sotorasib demonstrated an objective response rate (ORR) of 21%, median progression-free survival (mPFS) of 4.0 months, and median overall survival (mOS) of 6.9 months in previously treated KRAS G12C-mutated PDAC patients [30]. Similarly, adagrasib demonstrated encouraging activity in the KRYSTAL-1 study, reporting an ORR of 33% and disease control rate (DCR) of 81% in KRAS G12C-mutated pancreatic cancer [31]. Although these studies established proof-of-concept that KRAS-driven signaling in PDAC is therapeutically targetable, KRAS G12C mutations account for only approximately 1–2% of PDAC cases, substantially limiting the broader clinical applicability of these agents in pancreatic cancer [25].

Consequently, therapeutic development has increasingly shifted toward KRAS G12D, the predominant KRAS subtype in PDAC. Among emerging KRAS G12D-selective inhibitors, MRTX1133 demonstrated potent preclinical activity, inducing marked tumor regression and MAPK pathway suppression in both genetically engineered and patient-derived PDAC models [32,33]. Building upon these findings, several next-generation KRAS G12D inhibitors have rapidly entered early clinical development. Zoldonrasib (RMC-9805), a KRAS G12D-selective RAS(ON) inhibitor, demonstrated an ORR of 30% and DCR of 80% in KRAS G12D-mutated PDAC patients treated at 1200 mg/day while maintaining manageable tolerability profiles in an ongoing phase I/Ib study [34,35]. Similarly, INCB161734, an oral KRAS G12D ON/OFF inhibitor, demonstrated ORRs ranging from 23 to 34% with DCRs of 73–86% across evaluated dose levels, while combination studies with GnP and mFOLFIRINOX demonstrated encouraging feasibility without major dose-limiting toxicities [36,37]. Additional KRAS G12D-directed approaches including VS-7375 (GFH375), ASP3082, and HRS-4642 are also currently undergoing early clinical evaluation, further expanding the therapeutic landscape of direct KRAS inhibition.

Importantly, the clinical behavior of KRAS-targeted therapy in PDAC appears biologically distinct from that observed in KRAS-mutant lung cancer. Compared with NSCLC, PDAC exhibits substantially greater stromal restriction, metabolic plasticity, adaptive signaling redundancy, and immune suppression, all of which may attenuate the durability of KRAS inhibition despite initial pathway suppression. These observations suggest that effective KRAS targeting in PDAC may require broader suppression of adaptive survival networks beyond mutant KRAS blockade alone.

Accordingly, therapeutic development has increasingly expanded beyond allele-specific inhibition toward broader pan-RAS and RAS(ON)-targeting strategies. These agents aim to inhibit multiple KRAS variants or selectively target the active GTP-bound state of RAS proteins, thereby potentially overcoming mutation heterogeneity and adaptive signaling recovery across KRAS-driven tumors [11]. Among these agents, daraxonrasib (RMC-6236), a multi-selective RAS(ON) inhibitor, has emerged as one of the most promising therapeutic candidates in PDAC.

Early phase I/II studies demonstrated encouraging antitumor activity of daraxonrasib in previously treated RAS-mutant PDAC patients, supporting continued registrational development [38]. More recently, the global phase III RASolute 302 trial demonstrated significant survival benefit with daraxonrasib compared to standard chemotherapy in previously treated metastatic PDAC. Daraxonrasib achieved a median overall survival (mOS) of 13.2 months versus 6.7 months with chemotherapy, with a hazard ratio for death of 0.40 [39]. PFS and ORR were also substantially improved while maintaining a manageable safety profile. These findings represent one of the most clinically meaningful advances in KRAS-mutant PDAC to date and further validate KRAS-directed therapy as a viable therapeutic strategy in pancreatic cancer.

Nevertheless, accumulating evidence suggests that KRAS inhibition alone may be insufficient for durable disease control in PDAC. Extensive adaptive rewiring involving RTK/SHP2 reactivation, metabolic compensation, stromal-mediated protection, and immune suppression frequently restores tumor fitness despite suppression of mutant KRAS signaling. Consequently, current therapeutic development is increasingly shifting toward biologically rational combination strategies designed to simultaneously disrupt oncogenic signaling, adaptive resistance pathways, and the suppressive tumor microenvironment.

**Table 2:** Clinical Development of KRAS-Targeted Therapies in PDAC.

Agent	Molecular Target/Mechanism	Clinical Phase (N)	Key Findings in PDAC	Ref.
Sotorasib	KRAS G12C covalent inhibitor	Phase I/II (N = 38)	ORR 21%, mPFS 4.0 mo, mOS 6.9 mo in KRAS G12C PDAC	[30]
Adagrasib	KRAS G12C covalent inhibitor	Phase I/II (N = 64)	Among 21 patients with KRAS G12C-mutated PDAC, ORR was 33% and DCR was 81%	[31]
Zoldonrasib (RMC-9805)	KRAS G12D-selective RAS(ON) inhibitor	Phase I/Ib (N = 40)	ORR 30%, DCR 80% in KRAS G12D-mutated PDAC at 1200 mg/day; manageable tolerability	[34]
INCB161734	Oral KRAS G12D ON/OFF inhibitor	Phase I (All tumors N = 136; PDAC n = 83)	ORR 23–34%, DCR 73–86% across dose levels	[37]
Daraxonrasib (RMC-6236)	Multi-selective RAS(ON) inhibitor targeting multiple KRAS G12X/G13X/Q61X mutations	Phase III (N = 500)	In the overall population, mOS 13.2 vs. 6.7 months and mPFS 7.2 vs. 3.6 months, demonstrating significant survival benefit over chemotherapy	[39]
Daraxonrasib or Daraxonrasib + GnP	Pan-RAS inhibition combined with/without GnP	Phase III (ongoing)	1L combination therapy is currently under clinical evaluation, with preliminary activity reported.	[40]
Daraxonrasib + Vopimetostat	KRAS inhibition + PRMT5 synthetic lethality	Phase I/II (ongoing)	Clinical evaluation of this combination is ongoing in MTAP-deleted KRAS-mutant PDAC.	[41]
VS-7375 (GFH375)	Oral KRAS G12D ON/OFF inhibitor	Phase I/II	First-in-human dose-escalation study is ongoing	[42]
ASP3082	KRAS G12D degrader	Phase I	First-in-human dose-escalation study is ongoing	[43]
HRS-4642	KRAS G12D ON/OFF inhibitor	Phase I	Early safety and pharmacodynamic evaluation are ongoing	[44]
ERAS-4001	Pan-KRAS inhibitor	Preclinical	Next-generation pan-KRAS development entering clinical evaluation	[45]
MRTX1133	Selective KRAS G12D inhibitor	Preclinical	Potent tumor regression and immune modulation observed in PDAC models	[46]
SHP2 inhibitors	SHP2-mediated RTK bypass blockade	Preclinical	Delayed MAPK pathway reactivation and adaptive resistance	[47,48,49,50]

DCR, disease control rate; GnP, gemcitabine plus nab-paclitaxel; mOS, median overall survival; mPFS, median progression-free survival; ORR, objective response rate. Clinical phase indicates the highest publicly available stage of clinical development at the time of manuscript preparation. No peer-reviewed efficacy data are currently available for the daraxonrasib–vopimetostat combination; clinical evaluation remains ongoing.

### 4.3. Mechanisms of Resistance to KRAS Inhibition

Despite the remarkable progress in KRAS-targeted therapy, durable clinical responses remain constrained by the rapid emergence of adaptive and acquired resistance mechanisms. Increasing evidence indicates that KRAS inhibition in PDAC triggers extensive signaling reprogramming, allowing tumor cells to maintain survival and restore oncogenic output despite effective suppression of mutant KRAS activity [11].

One of the dominant adaptive mechanisms involves compensatory receptor tyrosine kinase (RTK) activation. Inhibition of KRAS signaling can induce feedback activation of EGFR, HER2, FGFR, and MET, resulting in reactivation of downstream MAPK and PI3K signaling cascades. SHP2 is a particularly important mediator of this process because it links activated RTKs to SOS1-dependent RAS nucleotide exchange and facilitates restoration of RAS pathway signaling following KRAS inhibition [47,48,49,50]. Thus, biochemical suppression of mutant KRAS may not be sufficient to maintain durable pathway inhibition when upstream signaling redundancy remains intact.

Acquired resistance may also arise through secondary genomic alterations. As observed in KRAS G12C-mutant lung cancer, secondary KRAS mutations, amplification of mutant KRAS alleles, and alterations involving downstream effectors such as BRAF, MEK, or PI3K may emerge under therapeutic pressure [50]. In addition, epithelial-to-mesenchymal transition and lineage plasticity can reduce dependence on canonical KRAS signaling and promote alternative survival states [51,52]. These findings support the concept that KRAS dependency in PDAC is dynamic and may be progressively attenuated during treatment.

Recent evidence further suggests that adaptive resistance to KRAS inhibition extends beyond canonical upstream or downstream signaling reactivation. Wang et al. demonstrated that YAP/TAZ activation functions as a central compensatory resistance mechanism following KRAS inhibitor exposure, contributing to both intrinsic and acquired resistance states [53]. Notably, KRAS inhibitor-resistant models exhibited cross-resistance to EGFR, SHP2, MEK, and PI3K inhibitors, suggesting that vertical blockade of the RAS signaling axis alone may be insufficient to fully overcome adaptive therapeutic escape. Mechanistically, YAP/TAZ activation suppressed the pro-apoptotic mediators BIM, BMF, and PUMA while simultaneously promoting SLC7A5-dependent mTORC1 reactivation, thereby sustaining both survival and proliferative recovery despite KRAS inhibition. Importantly, similar resistance mechanisms were also observed in KRAS G12D-driven pancreatic cancer models treated with MRTX1133, supporting Hippo–YAP/TAZ signaling as a broader adaptive resistance mechanism across KRAS-driven PDAC [53].

Beyond tumor-intrinsic signaling adaptation, tumor-extrinsic mechanisms also contribute substantially to resistance. Cancer-associated fibroblasts (CAFs), stromal cytokines, and immunosuppressive myeloid populations provide compensatory survival signals that attenuate the antitumor effects of KRAS blockade [54]. Concurrently, KRAS inhibition induces metabolic adaptation involving altered glutamine utilization, oxidative phosphorylation, autophagy, and redox homeostasis, thereby enabling tumor cells to maintain viability under therapeutic stress [55]. Unlike pathway reactivation, these mechanisms reflect broader adaptations of both the tumor and its surrounding microenvironment.

Taken together, resistance to KRAS inhibition in PDAC should be viewed as a dynamic biological process involving pathway reactivation, secondary genomic evolution, lineage plasticity, Hippo–YAP/TAZ activation, metabolic adaptation, and stromal support. These interconnected mechanisms explain why effective suppression of mutant KRAS does not necessarily translate into durable tumor control despite potent biochemical inhibition.

These findings underscore that the long-term clinical success of KRAS-targeted therapy will depend not only on achieving potent inhibition of mutant KRAS itself but also on preventing or overcoming the adaptive mechanisms that progressively restore tumor survival and growth. Consequently, considerable efforts have focused on developing rational KRAS-centered combination strategies designed to counteract these resistance pathways. The following section discusses these emerging therapeutic approaches.

### 4.4. Metabolic and Autophagy-Directed Targeting: Lessons from Translational Challenges

Beyond direct KRAS inhibition, therapeutic strategies targeting KRAS-driven metabolic dependencies have also attracted considerable interest in PDAC. Oncogenic KRAS reprograms multiple metabolic pathways, including glutamine utilization, macropinocytosis, autophagy, and oxidative phosphorylation, creating potential therapeutic vulnerabilities [56]. However, despite compelling biological rationale, clinical translation of metabolism-directed therapies has thus far produced inconsistent results.

One representative approach has been glutaminase inhibition using telaglenastat (CB-839), which was developed to exploit glutamine addiction in KRAS-driven tumors. Although early clinical studies demonstrated an acceptable safety profile, clinical activity remained modest, and meaningful efficacy has not yet been established in PDAC-specific populations [57]. Likewise, combination studies of telaglenastat with palbociclib in KRAS-mutant, CDKN2A-altered PDAC are ongoing, but definitive clinical benefit has not yet been demonstrated (NCT03965845).

Autophagy inhibition has produced similarly mixed clinical outcomes. In resectable PDAC, addition of high-dose hydroxychloroquine (HCQ) to gemcitabine plus nab-paclitaxel significantly improved histopathologic response and CA19-9 decline in a randomized phase II trial [58]. In contrast, a phase 2 randomized trial of 112 patients with metastatic PDAC found that adding HCQ to gemcitabine plus nab-paclitaxel did not improve the primary endpoint of overall survival at 12 months, despite a statistically significant increase in overall response rate from 21% to 38%, leading investigators to conclude that this regimen should not be used routinely in metastatic disease in the absence of a predictive biomarker, while noting that the response-rate improvement might still be relevant in the locally advanced setting where response could permit resection [59]. More recently, combined MEK and autophagy inhibition with trametinib plus HCQ achieved clinically meaningful disease stabilization in two reported cases of KRAS-mutated, chemoresistant PDAC, consistent with preclinical rationale, though prospective evidence remains unavailable [60].

Collectively, these studies illustrate an important translational lesson. Although metabolic and autophagy-directed therapies are strongly supported by PDAC biology, clinical efficacy appears highly dependent on disease stage, treatment context, and patient selection. In contrast to the recent randomized survival benefit observed with daraxonrasib, metabolism-directed approaches have not yet produced reproducible clinical benefit in PDAC. Future development will likely require functional biomarkers capable of identifying tumors with therapeutically exploitable metabolic vulnerabilities rather than treating metabolically heterogeneous PDAC as a single disease entity.

### 4.5. Combination Strategies with KRAS Inhibitors

Given the adaptive resistance mechanisms described above, combination strategies have emerged as a logical approach to prolong the efficacy of KRAS inhibition and delay pathway reactivation in PDAC.

One of the most actively investigated strategies involves co-targeting KRAS and SHP2 signaling. Because SHP2 functions as a critical mediator of RTK-driven RAS pathway reactivation, combined KRAS and SHP2 inhibition has demonstrated synergistic antitumor activity in preclinical PDAC models [49]. Similarly, combinations involving EGFR, MEK, or PI3K inhibitors are being explored to suppress bypass signaling and downstream pathway recovery following KRAS blockade. Collectively, these approaches aim to maintain durable suppression of RAS pathway signaling by preventing adaptive feedback activation.

Combination approaches integrating KRAS inhibitors with cytotoxic chemotherapy also represent an important area of clinical development. Chemotherapy-induced stress may transiently increase tumor dependency on KRAS signaling while partially destabilizing adaptive survival states, thereby enhancing sensitivity to KRAS-targeted therapy [11]. Consequently, these combinations are being investigated not only to increase cytotoxic efficacy but also to delay the emergence of adaptive resistance.

More recently, synthetic lethal strategies have emerged as particularly promising KRAS-centered therapeutic approaches. Among these, the combination of daraxonrasib with the PRMT5 inhibitor vopimetostat has generated considerable interest in MTAP-deleted PDAC. MTAP deletion creates a selective dependency on PRMT5 signaling, thereby establishing a biologically rational synthetic lethal interaction that complements KRAS inhibition [61]. Preliminary clinical findings demonstrated encouraging antitumor activity of daraxonrasib combined with vopimetostat in MTAP-deleted RAS-mutant PDAC, supporting further clinical evaluation of biomarker-selected synthetic lethal strategies [41].

Collectively, these studies indicate that current KRAS-centered combination strategies primarily seek to suppress adaptive pathway restoration or exploit vulnerabilities that become apparent following KRAS inhibition. These approaches remain mechanistically focused on overcoming KRAS-specific resistance and therefore differ from broader cross-mechanism therapeutic frameworks discussed later in this review.

## 5. DDR-Targeted Therapeutic Strategies and Beyond-BRCA Approaches

### 5.1. DNA Damage Response Alterations in PDAC

Defects in the DDR pathway have emerged as important therapeutic vulnerabilities in PDAC. Although PDAC is generally characterized by a relatively low prevalence of actionable genomic alterations, approximately 15–25% of cases harbor mutations involving homologous recombination repair (HRR)-related genes, including BRCA1, BRCA2, PALB2, ATM, CHEK2, and RAD51-associated pathways [19,62]. These alterations impair DNA double-strand break repair and increase genomic instability, thereby creating potential susceptibility to synthetic lethal therapeutic strategies.

Among DDR alterations, germline BRCA1/2 mutations represent the most clinically validated subgroup in PDAC. BRCA-mutated tumors exhibit impaired homologous recombination repair and increased sensitivity to platinum-based chemotherapy and PARP inhibition [63]. In addition to BRCA1/2, alterations involving PALB2 and ATM have also been associated with defective DNA repair and may confer varying degrees of DDR-targeted therapeutic sensitivity [19].

Importantly, recent evidence suggests that DDR vulnerability in PDAC extends beyond germline BRCA mutations alone. The broader concept of HRD has emerged as a clinically relevant framework encompassing genomic instability, replication stress, and functional impairment of DNA repair pathways [64]. However, defining HRD in PDAC remains challenging because genomic mutations do not always correlate with functional DDR deficiency or therapeutic responsiveness.

Beyond genomic instability, DDR signaling also contributes to tumor progression and immune modulation. Accumulation of unrepaired DNA damage may activate innate immune pathways such as cGAS-STING signaling, potentially enhancing interferon responses, neoantigen generation, and antitumor immunity [65]. These findings have generated increasing interest in therapeutic strategies combining DDR-targeted agents with immunotherapy and KRAS inhibition in PDAC.

Collectively, DDR alterations represent one of the most clinically relevant precision oncology opportunities in PDAC and provide a strong biological rationale for synthetic lethal therapeutic development.

### 5.2. Clinical Development of PARP Inhibitors

PARP inhibitors represent the first clinically successful biomarker-driven targeted therapy strategy in PDAC. By exploiting synthetic lethality in tumors with defective homologous recombination repair, PARP inhibition selectively induces accumulation of unrepaired DNA damage, ultimately leading to tumor cell death [66].

The most important clinical milestone in this field was the phase III POLO trial, which evaluated maintenance olaparib in patients with germline BRCA-mutated metastatic PDAC whose disease had not progressed during first-line platinum-based chemotherapy [19]. Olaparib significantly prolonged median progression-free survival compared with placebo (7.4 vs. 3.8 months), establishing PARP inhibition as the first biomarker-selected maintenance strategy in PDAC. Although overall survival benefit was less definitive, the study demonstrated the clinical feasibility of precision oncology approaches in pancreatic cancer.

Following the POLO trial, multiple PARP inhibitors including olaparib, rucaparib, niraparib, talazoparib, and veliparib have been evaluated in PDAC across maintenance, combination, and later-line settings [67]. Among these agents, veliparib demonstrated limited efficacy as monotherapy but showed potential synergy when combined with platinum-based chemotherapy or radiotherapy, although toxicity remained a major limitation [68].

More recently, therapeutic development has increasingly shifted beyond germline BRCA-mutated populations toward broader DDR-deficient and HRD-positive subgroups. However, clinical activity of PARP inhibitors in non-BRCA DDR-altered PDAC has generally been more modest and heterogeneous than initially anticipated [64]. These findings highlight the complexity of DDR biology in PDAC and underscore the need for improved biomarkers capable of identifying functional HRD and predicting PARP inhibitor sensitivity.

To overcome resistance and expand therapeutic applicability, current strategies are increasingly exploring PARP inhibitor-based combination approaches. Preclinical and early clinical studies have demonstrated potential synergy between PARP inhibitors and multiple therapeutic strategies, including ATR, WEE1, and CHK1 inhibition, as well as chemotherapy, KRAS-targeted therapy, and immune checkpoint blockade, supporting the development of rational combination approaches in pancreatic cancer [69,70,71,72,73]. In particular, dual-targeting strategies combining PARP inhibition with immune modulation or replication stress induction may represent promising approaches for overcoming adaptive resistance in PDAC.

Collectively, PARP inhibitors have established proof-of-concept for DDR-directed precision oncology in PDAC, while simultaneously highlighting the broader need for more effective biomarker-guided and combination-based therapeutic strategies.

Importantly, the therapeutic paradigm of DDR targeting in PDAC is gradually shifting from static genomic classification toward therapeutically induced vulnerability. Rather than relying solely on pre-existing BRCA mutations, emerging strategies increasingly aim to pharmacologically induce “BRCAness,” replication stress, or synthetic lethal dependency in genomically broader PDAC populations [74]. Accordingly, recent therapeutic development has increasingly expanded beyond canonical BRCA-associated disease toward broader concepts of functional HRD, replication stress targeting, and synthetic lethality.

### 5.3. Expanding Beyond BRCA-Driven Therapeutic Strategies

Although germline BRCA1/2-mutated PDAC represents the most clinically validated subgroup for PARP inhibitor therapy, increasing evidence suggests that DDR-targeted therapeutic vulnerability extends beyond canonical BRCA-associated alterations alone. Consequently, recent therapeutic development has increasingly shifted from exploiting pre-existing HRD toward therapeutically inducing “BRCAness” and replication stress vulnerability in BRCA-proficient tumors (Table 3).

Multiple non-BRCA DDR-related alterations, including PALB2, ATM, CHEK2, RAD51, and Fanconi anemia pathway genes, have been implicated in defective DNA repair and genomic instability in PDAC [62]. However, clinical responses to PARP inhibition among these subgroups have generally been heterogeneous and less robust than those observed in BRCA-mutated disease, highlighting the limitations of mutation-based patient selection alone [64]. Accordingly, current efforts are increasingly focused on identifying functional HRD phenotypes and therapeutically inducible DDR vulnerabilities.

Replication stress has emerged as a particularly important therapeutic target in KRAS-driven PDAC. Constitutive oncogenic KRAS signaling promotes uncontrolled proliferation, oxidative stress, and DNA replication instability, thereby increasing dependency on ATR-, CHK1-, and WEE1-mediated checkpoint signaling [70,71]. Inhibition of these pathways may selectively sensitize tumor cells to DNA damage accumulation and mitotic catastrophe, particularly in tumors exhibiting high replication stress burden.

Among emerging DDR-directed approaches, nesuparib (JPI-547), a dual inhibitor of tankyrase 1/2 (TNKS1/2) and PARP1/2, represents a strategy aimed at extending DDR-targeted therapy beyond conventional BRCA-mutated populations. Unlike conventional PARP inhibitors, nesuparib was designed to induce a “BRCAness” phenotype through simultaneous inhibition of PARP-mediated DNA repair and TNKS-dependent signaling pathways, including WNT/β-catenin and Hippo signaling [75]. By suppressing adaptive WNT- and KRAS-associated signaling while impairing homologous recombination repair, dual TNKS/PARP inhibition may broaden synthetic lethal vulnerability in BRCA wild-type PDAC.

Preclinical studies further demonstrated that nesuparib enhanced the antitumor efficacy of gemcitabine plus nab-paclitaxel (GnP) in BRCA wild-type pancreatic cancer xenograft models [76]. In MIA PaCa-2 models, the addition of nesuparib increased tumor growth inhibition from 31% with GnP alone to 79% with combination treatment. Mechanistically, nesuparib suppressed compensatory upregulation of c-Myc, MYBL2, β-catenin, cyclin D1, and RAD51, suggesting that dual TNKS/PARP inhibition may simultaneously impair DNA repair capacity while attenuating WNT-associated adaptive resistance and KRAS-driven compensatory signaling rewiring. Notably, adaptive resistance to KRAS inhibition has been associated with YAP/TAZ-mediated lineage plasticity, suppression of pro-apoptotic signaling, and metabolic rewiring through the SLC7A5/mTOR axis [53]. In this context, TNKS inhibition may provide therapeutic benefit beyond conventional DDR targeting by modulating Hippo-YAP/TAZ signaling and adaptive tumor plasticity. Consistent with this concept, additional preclinical studies demonstrated stabilization of AMOT proteins, increased phospho-YAP, and suppression of WNT/Hippo signaling following nesuparib treatment [77,78]. Collectively, these findings suggest that dual TNKS/PARP inhibition may function not only as a DDR-directed strategy but also as an approach to suppress adaptive signaling rewiring associated with KRAS-driven tumors.

An ongoing multicenter phase Ib/II study is evaluating nesuparib in combination with GnP or modified FOLFIRINOX (mFOLFIRINOX) in patients with locally advanced or metastatic PDAC [79]. In the GnP cohort, preliminary results showed an ORR of 53.8% and a DCR of 92.3%, with a median OS of 14.2 months at an immature data cutoff. Although these findings require validation in randomized studies, they support further investigation of dual TNKS/PARP inhibition as a potential strategy for expanding DDR-directed therapy beyond conventional HRD-positive population.

In parallel, additional synthetic lethal strategies are also rapidly emerging in PDAC. One notable example involves PRMT5 inhibition in MTAP-deleted tumors. MTAP deletion, which occurs in approximately 10–15% of PDAC cases, creates a metabolic dependency on PRMT5 signaling, thereby generating a potentially targetable synthetic lethal vulnerability [41,61]. Consistent with this rationale, several next-generation PRMT5 inhibitors are currently undergoing clinical evaluation, including TNG462, which is being investigated in combination with standard chemotherapy and KRAS-targeted agents in patients with MTAP-deleted PDAC (NCT06922591). Collectively, these developments indicate that future DDR-targeted therapeutic strategies in PDAC will likely expand beyond static genomic classification toward broader concepts of functional HRD, induced “BRCAness,” replication stress, and synthetic lethality.

**Table 3:** DDR-Targeted Therapeutic Strategies in PDAC.

Agent	Mechanism	Target Population	Combination Partner	Development Stage	Key Findings	Ref.
Olaparib	PARP inhibitor	Germline BRCA-mutated PDAC	-	Phase III (POLO, N = 154)	mPFS 7.4 vs. 3.8 months as maintenance therapy	[19]
TNG462 (vopimetostat)	MTA-cooperative PRMT5 inhibitor	MTAP-deleted PDAC	KRAS inhibitors/ chemotherapy	Phase I/II (ongoing)	Ongoing clinical evaluation; combination studies in MTAP-deleted PDAC are underway	[41]
Veliparib	PARP inhibitor	BRCA/PALB2-mutated PDAC	Cisplatin + gemcitabine	Phase II (N = 50)	Improved response rate without significant overall survival benefit	[68]
Nesuparib (JPI-547)	Dual TNKS/PARP inhibitor	Locally advanced and metastatic PDAC	Gemcitabine + nab-paclitaxel	Phase Ib/II (N = 26)	ORR 53.8%, DCR 92.3%, mOS 14.2 months (immature)	[79]
AZD6738 (ceralasertib)	ATR inhibitor	PDAC	Gemcitabine	Preclinical	Synergized with gemcitabine in PDAC models	[80]
Adavosertib (AZD1775)	WEE1 inhibitor	Locally advanced PDAC	Gemcitabine + radiotherapy	Phase I (N = 34)	mOS 21.7 months and mPFS 9.4 months with manageable safety	[81]

ORR, objective response rate; DCR, disease control rate; mPFS, median progression-free survival; mOS, median overall survival. Results for nesuparib are based on an ongoing phase Ib/II study with immature survival data. TNG462 is currently under clinical evaluation, and peer-reviewed efficacy results are not yet available.

### 5.4. Interplay Between DDR Signaling and Antitumor Immunity

Beyond its canonical role in maintaining genomic integrity, DNA damage response (DDR) signaling has emerged as an important regulator of antitumor immunity. Accumulation of unrepaired DNA damage may increase cytosolic DNA fragments, thereby activating the cyclic GMP–AMP synthase–stimulator of interferon genes (cGAS–STING) pathway [65]. Activation of this innate immune sensing pathway promotes type I interferon production, dendritic cell maturation, and T-cell priming, thereby increasing the immunogenic potential of tumor cells. Consistent with these observations, preclinical studies have demonstrated that PARP inhibition can enhance antitumor immunity through multiple mechanisms, including increased neoantigen generation, activation of interferon signaling, and upregulation of PD-L1 expression [82]. These findings suggest that DDR-directed therapy may influence not only tumor cell survival but also the immunologic properties of the tumor. However, translating these immunostimulatory effects into meaningful clinical benefit remains particularly challenging in PDAC. Although DDR inhibition may increase tumor immunogenicity, the profoundly immunosuppressive microenvironment characteristic of PDAC continues to limit effective immune activation. Extensive stromal fibrosis, myeloid-mediated immune suppression, and poor T-cell infiltration remain major barriers that prevent innate immune activation from generating durable antitumor responses [7]. Accordingly, one of the key unresolved questions is whether DDR-induced immunogenicity can be effectively converted into sustained adaptive immune responses in patients with PDAC. Addressing this question will require a better understanding of the relationship between DNA damage signaling, innate immune activation, and the unique stromal ecosystem of pancreatic cancer.

## 6. Immunotherapy and Tumor Microenvironment Reprogramming in PDAC

### 6.1. Why Immune Checkpoint Inhibitors Fail in PDAC

Despite the transformative success of immune checkpoint inhibitors (ICIs) in multiple solid tumors, their efficacy in PDAC has remained remarkably limited. Except for the rare subset of microsatellite instability-high (MSI-H) or mismatch repair-deficient tumors, single-agent PD-1/PD-L1 or CTLA-4 blockade has demonstrated minimal clinical benefit in PDAC [7,83].

One of the primary reasons for immunotherapy resistance in PDAC is its profoundly immunosuppressive tumor microenvironment. PDAC is characterized by extensive desmoplastic fibrosis and dense stromal remodeling, which create both physical and biochemical barriers to effective T-cell infiltration [9]. CAFs, extracellular matrix deposition, and abnormal tumor vasculature collectively contribute to immune exclusion and impaired intratumoral immune trafficking.

In addition, PDAC exhibits a highly suppressive myeloid-dominant immune landscape enriched with tumor-associated macrophages, myeloid-derived suppressor cells (MDSCs), and regulatory T cells (Tregs) [7]. These cell populations inhibit cytotoxic T-cell activation through secretion of immunosuppressive cytokines including TGF-β, IL-10, and CXCL12, thereby promoting immune tolerance and resistance to checkpoint blockade.

Tumor-intrinsic biology further contributes to immune evasion. Oncogenic KRAS signaling promotes the secretion of immunosuppressive cytokines and chemokines while simultaneously suppressing antigen presentation and dendritic cell recruitment [28]. Moreover, PDAC generally exhibits a relatively low tumor mutational burden and limited neoantigen generation compared to immunotherapy-responsive tumors such as melanoma or lung cancer, reducing baseline tumor immunogenicity [3].

Clinical studies have consistently reflected these biological barriers. Early trials evaluating ipilimumab or PD-1 blockade in unselected PDAC populations demonstrated extremely limited objective responses and poor survival outcomes [83]. Even combination checkpoint strategies have thus far produced only modest activity, emphasizing that immune checkpoint inhibition alone is insufficient to overcome the complex immunosuppressive network characteristic of PDAC.

Importantly, PDAC should not simply be viewed as an immunologically “cold” tumor, but rather as an actively immune-excluded ecosystem in which stromal architecture, myeloid dominance, oncogenic KRAS signaling, metabolic adaptation, and suppressive cytokine networks cooperatively limit effective antitumor immunity. This biological complexity may partially explain why conventional checkpoint inhibition alone has consistently failed to generate durable clinical benefit in PDAC. Consequently, successful immunotherapy in PDAC will likely require multidimensional immune remodeling strategies capable of simultaneously enhancing T-cell infiltration, reversing myeloid-mediated suppression, and increasing tumor immunogenicity beyond conventional checkpoint blockade alone.

### 6.2. Surface-Targeted Immune-Redirection Strategies in PDAC

Given the limited efficacy of conventional immune checkpoint blockade in PDAC, recent immunotherapeutic development has increasingly shifted toward surface-targeted immune-redirection strategies designed to actively recruit and activate cytotoxic immune responses within the highly suppressive tumor microenvironment. Unlike checkpoint inhibitors, which largely depend on pre-existing endogenous antitumor immunity, these approaches directly redirect immune effector cells toward tumor-associated antigens and may therefore partially overcome the limited spontaneous T-cell infiltration characteristic of PDAC.

Among the most actively investigated platforms are T-cell engagers (TCEs), bispecific antibodies, antibody–drug conjugates (ADCs), and chimeric antigen receptor (CAR)-T cell therapies. These modalities target tumor-associated surface antigens including mesothelin, Claudin18.2 (CLDN18.2), carcinoembryonic antigen (CEA), EGFR, HER2, MUC1, and fibroblast activation protein (FAP), many of which are highly expressed in PDAC while demonstrating relatively restricted expression in normal tissues [84].

Mesothelin represents one of the earliest and most extensively investigated targets in PDAC. Multiple mesothelin-directed therapeutic platforms, including CAR-T cells, vaccines, ADCs, and bispecific antibodies, have demonstrated preliminary antitumor activity in early-phase clinical studies [85]. However, durable therapeutic efficacy remains limited because of antigen heterogeneity, restricted T-cell persistence, stromal exclusion, and adaptive immune resistance within the PDAC microenvironment.

More recently, CLDN18.2-directed therapies have emerged as one of the most promising immune-redirection strategies in gastrointestinal malignancies, including PDAC. CLDN18.2 is normally restricted to differentiated gastric mucosal epithelial cells but becomes aberrantly exposed and retained in several gastrointestinal malignancies, making it an attractive therapeutic target [86]. Clinical success of zolbetuximab in gastric and gastroesophageal cancers established proof-of-concept for CLDN18.2-targeted therapy in solid tumors [87], leading to rapid expansion of next-generation CLDN18.2-targeted platforms in PDAC.

Representative agents currently under investigation include givastomig (AZD5863), IBI389, QLS31905, and multiple CLDN18.2-directed bispecific T-cell engager platforms designed to enhance immune-cell recruitment and cytotoxic activation within immunologically “cold” tumors. In parallel, CLDN18.2-directed CAR-T therapies such as satricabtagene autoleucel (CT041) have demonstrated encouraging early clinical activity in heavily pretreated gastrointestinal cancers, including durable responses in selected patients [88]. Among emerging bispecific approaches, spevatamig (PT886), a CLDN18.2/CD47 bispecific antibody, has demonstrated encouraging preliminary activity in combination with gemcitabine plus nab-paclitaxel in CLDN18.2-positive metastatic PDAC [89]. Similarly, preliminary results from an early-phase study of the CLDN18.2/CD3 bispecific T-cell engager QLS31905 demonstrated promising efficacy in combination with chemotherapy for first-line CLDN18.2-positive PDAC, with median progression-free survival and overall survival of 8.7 and 15.9 months, respectively [90].

Despite these encouraging clinical results, CLDN18.2-directed therapy may also encounter adaptive resistance mechanisms. In gastric cancer, longitudinal analyses have demonstrated treatment-associated conversion from CLDN18.2-positive to CLDN18.2-negative status following zolbetuximab exposure, with 53.3% of evaluable patients converting to CLDN18.2-negative status (using a ≥75% cut-off) at disease progression, suggesting that antigen loss may represent an important mechanism of therapeutic escape [91]. In PDAC, a study comparing 211 resected tumors with 133 matched biopsies reported 92.5% concordance at the standard 75% threshold; however, this figure largely reflected the high proportion of CLDN18.2-negative cases, as biopsy sensitivity for truly positive tumors was only 54.6%, raising concern that biopsy-based assessment may underdiagnose eligible patients [92]. Lowering the threshold to 20% improved sensitivity to 100%, but patients meeting only this lower threshold would still be excluded under current trial eligibility criteria, illustrating a clinical dilemma between sensitivity and trial-defined cutoffs. CLDN18.2 expression was largely preserved in recurrent lesions (83.3% concordance), though reductions were noted in local recurrence and liver metastasis. Together, these findings indicate that CLDN18.2-targeted therapy will require both improved baseline biopsy strategies and longitudinal reassessment of antigen expression over the course of treatment.

Beyond direct tumor-cell targeting, stromal-associated surface antigens are also emerging as therapeutic targets to address the physical and immunosuppressive barriers imposed by the desmoplastic PDAC microenvironment. FAP-directed CAR-T cells, for example, have been shown to deplete cancer-associated fibroblasts and suppress myeloid-derived suppressor cell recruitment, thereby enhancing the subsequent antitumor efficacy of CLDN18.2-targeted CAR-T cells when administered sequentially in preclinical PDAC models [93]. In parallel, EGFR- and HER2-directed bispecific T-cell engagers are being explored to broaden the repertoire of targetable surface antigens: heterodimeric EGFR × HER2 constructs have demonstrated selective cytotoxicity against EGFR/HER2 double-positive PDAC xenografts while sparing single-positive tissue, offering a potential strategy to mitigate on-target/off-tumor toxicity while addressing antigen heterogeneity across tumor subpopulations [94].

Despite this expanding target repertoire, effective immune redirection in PDAC remains constrained less by antigen availability than by the tumor microenvironment itself. Durable antitumor activity depends not only on successful antigen recognition but on efficient immune-cell trafficking into the desmoplastic stroma, sustained effector-cell persistence, and preservation of cytotoxic function against a background of chronic antigen exposure and myeloid-driven suppression. These barriers have limited clinical responses even when antigen engagement itself is robust, as seen with the mesothelin- and CLDN18.2-directed platforms discussed above [95].

Collectively, current evidence indicates that surface-targeted immune-redirection platforms can partially overcome the limited endogenous antitumor immunity characteristic of PDAC by directly engaging immune effector cells against tumor and stromal antigens. However, translating antigen engagement into durable clinical benefit will depend on resolving the trafficking, persistence, and toxicity barriers outlined above, alongside the antigen-loss and expression-heterogeneity concerns discussed for CLDN18.2 in the preceding section. As discussed further in Section 7, these challenges are themselves rooted in the same stromal signaling network shared across KRAS-, DDR-, and immune-directed therapeutic strategies in PDAC.

### 6.3. Tumor Microenvironment Remodeling Strategies

The TME of PDAC represents one of the principal drivers of therapeutic resistance and immune evasion. PDAC is characterized by extensive desmoplastic fibrosis, hypovascularity, elevated interstitial pressure, and profound immune suppression, collectively impairing drug delivery and limiting effective antitumor immunity [10]. Consequently, recent therapeutic development has increasingly focused on active tumor microenvironment remodeling strategies aimed at converting immunologically “cold” tumors into more treatment-responsive states.

CAFs are central regulators of stromal remodeling in PDAC. CAF-derived extracellular matrix proteins, including collagen and hyaluronic acid, generate dense fibrotic architecture that restricts penetration of cytotoxic agents and immune cells [9]. In addition, CAF-secreted cytokines and chemokines promote recruitment of immunosuppressive myeloid populations, further reinforcing immune exclusion.

Initial stromal depletion strategies generated considerable enthusiasm; however, genetic ablation of myofibroblasts and Sonic Hedgehog-deficient stroma in preclinical PDAC models unexpectedly resulted in more invasive, poorly differentiated tumors and reduced survival, indicating that certain stromal components restrain rather than support tumor progression [96]. These findings shifted the field away from indiscriminate stromal depletion toward more selective stromal normalization and immune-reprogramming approaches.

One of the earliest clinically advanced stromal-targeting approaches involved pegvorhyaluronidase alfa (PEGPH20), a hyaluronidase-based therapy developed to degrade hyaluronic acid and improve intratumoral perfusion. Early studies demonstrated improved drug delivery and progression-free survival in hyaluronan-high PDAC subsets; however, the subsequent phase III HALO-301 trial failed to demonstrate overall survival benefit when PEGPH20 was combined with GnP [97]. Despite its ultimate failure, PEGPH20 provided important proof-of-concept supporting the biological relevance of stromal modulation in PDAC.

More recently, immune microenvironment remodeling strategies have emerged as particularly promising therapeutic approaches. Among these, targeting the CXCR4/CXCL12 axis has generated substantial interest because CAF-derived CXCL12 signaling contributes to T-cell exclusion and immune suppression within PDAC tumors. In the phase II COMBAT trial, the CXCR4 inhibitor motixafortide (BL-8040) combined with pembrolizumab and chemotherapy demonstrated encouraging immune activation and preliminary clinical activity in metastatic PDAC, including increased CD8-positive T-cell infiltration within the tumor microenvironment [98]. These findings provided clinical evidence that TME-directed immune remodeling may partially sensitize PDAC to immunotherapy.

CD40 agonists have also emerged as important immune-reprogramming strategies in PDAC. CD40 activation promotes dendritic cell maturation, macrophage reprogramming, and enhanced T-cell priming within the suppressive tumor microenvironment [99]. In early clinical studies, the CD40 agonist sotigalimab combined with chemotherapy and nivolumab demonstrated encouraging immune activation and survival signals in metastatic PDAC patients [100]. Similarly, neoadjuvant studies evaluating selicrelumab demonstrated increased T-cell infiltration and macrophage remodeling within resectable PDAC tumors, supporting the biological potential of CD40-mediated TME reprogramming [101].

Targeting myeloid-mediated immune suppression has also become an important therapeutic strategy. PDAC tumors are enriched with tumor-associated macrophages (TAMs) and MDSCs, both of which contribute to checkpoint inhibitor resistance and immune exclusion. Consequently, CSF1R inhibition has been investigated as a strategy to deplete suppressive macrophage populations and enhance antitumor immunity. Early studies evaluating cabiralizumab, a CSF1R inhibitor, in combination with nivolumab demonstrated preliminary clinical activity and evidence of macrophage modulation in advanced PDAC [102].

In parallel, focal adhesion kinase (FAK) inhibition and TGF-β-targeting approaches are also being actively explored as stromal normalization strategies. FAK signaling contributes to stromal stiffness, fibrosis, and immune exclusion, while TGF-β signaling promotes CAF activation and suppressive immune remodeling. Although clinical efficacy has thus far remained modest, these pathways continue to represent important therapeutic targets within integrated TME-modulating combination strategies.

Importantly, recent evidence suggests that tumor-intrinsic targeted therapies may themselves induce partial microenvironment remodeling. KRAS inhibition has been shown to alter cytokine signaling and enhance immune-cell infiltration, while DDR-targeted therapies may activate cGAS-STING-mediated innate immune signaling and increase tumor immunogenicity [65]. Consequently, current therapeutic development increasingly focuses on integrated combination approaches capable of simultaneously targeting oncogenic signaling, stromal barriers, and immune suppression.

Collectively, these findings suggest that effective immunotherapy in PDAC will likely require coordinated remodeling of multiple interconnected resistance networks rather than isolated immune activation alone. Future therapeutic strategies may therefore depend on integrated approaches capable of simultaneously targeting stromal restriction, myeloid-mediated suppression, oncogenic signaling, and adaptive metabolic programs within the PDAC microenvironment.

### 6.4. Cancer Vaccine Approaches: From Repeated Clinical Failure to Precision Immunization

Cancer vaccines represent one of the earliest immunotherapeutic strategies explored in PDAC, yet their clinical development has been characterized by repeated discrepancies between promising immunogenicity and limited survival benefit. Early whole-cell vaccine platforms, including GVAX and algenpantucel-L, generated encouraging immune responses and favorable early-phase survival signals [103,104,105]. However, algenpantucel-L subsequently failed to improve overall survival in two independent randomized phase III trials, in both the resected and the borderline resectable/locally advanced settings, while GVAX combined with CRS-207 was similarly discontinued after a phase 2b trial failed to demonstrate survival benefit, substantially limiting enthusiasm for these non-personalized vaccine approaches [15].

More recently, advances in genomic sequencing have enabled individualized neoantigen-based mRNA vaccines. In a phase I study, autogene cevumeran administered together with atezolizumab and modified FOLFIRINOX induced robust neoantigen-specific T-cell responses in half of treated patients, and vaccine responders demonstrated prolonged recurrence-free survival during long-term follow-up [106,107]. These findings provide compelling proof-of-concept that personalized vaccination may overcome some limitations of earlier vaccine platforms.

Beyond individualized vaccines, shared mutant KRAS-directed vaccines have recently emerged as an attractive off-the-shelf strategy. A phase I study demonstrated that a pooled synthetic long-peptide vaccine targeting multiple KRAS mutations, combined with dual immune checkpoint blockade, induced strong KRAS-specific T-cell responses in most treated patients with resected PDAC [108]. Nevertheless, enthusiasm has recently been tempered by the phase 2 AMPLIFY-7P trial of ELI-002 7P, which did not meet its pre-specified primary endpoint of disease-free survival (DFS) in the intent-to-treat population, despite demonstrating strong biological activity, with mutant KRAS-specific T-cell responses significantly correlating with improved DFS [109].

Taken together, these observations highlight a recurring pattern in PDAC vaccine development. Across whole-cell vaccines, personalized neoantigen vaccines, and shared KRAS-directed vaccines, early induction of robust antitumor immunity has not consistently translated into durable survival benefit in adequately powered randomized trials. Future vaccine development will therefore require improved predictive biomarkers, optimized combination strategies, and rational integration with therapies capable of simultaneously remodeling the immunosuppressive tumor microenvironment. The representative therapeutic platforms discussed across Section 6.2, Section 6.3 and Section 6.4 are summarized in Table 4.

**Table 4:** Emerging Immunotherapy and Tumor Microenvironment Remodeling Strategies in PDAC.

Strategy	Representative Agents/Platforms	Target	Development Stage	Clinical Findings	Ref.
Mesothelin- targeted immunotherapy	Mesothelin CAR-T, ADCs, vaccines	Mesothelin- directed immune targeting	Early clinical	Preliminary activity observed, but limited durability because of TME-mediated resistance	[85]
CLDN18.2/CD47 bispecific antibody	Spevatamig (PT886)	CLDN18.2 + innate immune activation via CD47 blockade	Phase I/II (TWINPEAK, N = 54)	mPFS 7.3 mo, mOS 13.2 mo	[89]
CLDN18.2/CD3 T-cell engager	QLS31905	T-cell redirection toward CLDN18.2-positive tumors	Phase Ib/II (N = 82)	mPFS 8.74 mo, mOS 15.87 mo	[90]
CLDN18.2 CAR-T therapy	Satricabtagene autoleucel (CT041)	CLDN18.2-targeted CAR-T	Phase I (N = 98)	mPFS 4.4 mo, mOS 8.4 mo across treated patients; 10 of 98 patients had pancreatic cancer.	[91]
Hyaluronidase-based stromal remodeling	PEGPH20	Hyaluronic acid degradation and stromal decompression	Phase III (HALO-301, N = 494)	Improved perfusion but failed to demonstrate OS benefit	[97]
CXCR4 inhibition	Motixafortide (BL-8040)	CXCR4/CXCL12 axis blockade	Phase II (COMBAT, N = 43)	Increased CD8-positive T-cell infiltration and encouraging immune activation with pembrolizumab plus chemotherapy	[98]
CD40 agonist therapy	Sotigalimab, Selicrelumab	APC activation and macrophage reprogramming	Early clinical	Enhanced T-cell priming and immune remodeling signals in metastatic and neoadjuvant PDAC studies	[99,100,101]
CSF1R inhibition	Cabiralizumab	Tumor-associated macrophage depletion	Early clinical	Preliminary macrophage modulation and immune activation observed with nivolumab combinations	[103]
FAK inhibition	Defactinib	Stromal stiffness, fibrosis modulation, and immune microenvironment remodeling	Phase I (N = 20)	Combination with pembrolizumab and gemcitabine demonstrated manageable safety and preliminary immune activation	[110]
TGF-β pathway inhibition	Galunisertib	Fibrosis and immune suppression targeting	Phase Ib/II (N = 32)	Combination with durvalumab demonstrated acceptable safety and modest clinical activity	[111]

ADC, antibody–drug conjugate; APC, antigen-presenting cell; CAR-T, chimeric antigen receptor T cell; CD, cluster of differentiation; CLDN18.2, claudin 18.2; COMBAT, Combination of BL-8040, pembrolizumab, and chemotherapy trial; CXCR4, C-X-C chemokine receptor type 4; mOS, median overall survival; mPFS, median progression-free survival; PDAC, pancreatic ductal adenocarcinoma; TCE, T-cell engager; TGF-β, transforming growth factor-β; TME, tumor microenvironment. Clinical outcomes from early-phase studies should be considered preliminary. Cross-trial comparisons are not appropriate because of differences in treatment line, biomarker definitions, patient selection, sample size, and follow-up duration.

## 7. Cross-Mechanism Integration and Future Therapeutic Framework

Recent advances in PDAC therapeutics indicate that KRAS-targeted therapy, DDR-directed therapy, and immune-redirection strategies should no longer be regarded as independent therapeutic classes. Instead, these modalities represent complementary approaches that address distinct biological processes underlying adaptive therapeutic resistance. This evolving perspective shifts the focus from targeting isolated molecular alterations toward disrupting the interconnected biological networks that enable PDAC to maintain tumor fitness despite therapeutic intervention [8,112].

A central concept emerging from recent translational research is that treatment failure in PDAC is driven less by incomplete inhibition of a single oncogenic pathway than by rapid systems-level adaptation. Although KRAS inhibition effectively suppresses the dominant oncogenic driver, tumors rapidly restore proliferative capacity through compensatory receptor tyrosine kinase (RTK) signaling, SHP2-dependent feedback activation, metabolic reprogramming, lineage plasticity, and stromal-derived survival cues [8,11,48,58]. Importantly, these adaptive mechanisms are increasingly recognized across multiple therapeutic modalities, highlighting adaptive plasticity as a defining hallmark of PDAC treatment resistance [112].

Rather than functioning solely as treatments for tumors harboring specific genomic alterations, DDR-directed therapies may also exploit biological vulnerabilities that arise during adaptive resistance. Increased replication stress and altered DNA repair dependency following therapeutic pressure create opportunities for synthetic lethal interventions involving PARP, ATR, WEE1, or PRMT5 inhibition [66,113]. Likewise, dual TNKS/PARP inhibition has been shown to simultaneously modulate WNT/β-catenin signaling, Hippo signaling, and DNA repair pathways, illustrating how network-level modulation may generate broader therapeutic benefit than mutation-restricted DDR approaches [71,72,73]. Collectively, these observations support a more dynamic view of precision oncology based on treatment-induced biological states rather than static genomic alterations.

A similar conceptual evolution is occurring in cancer immunotherapy. Surface-targeted immune-redirection platforms, including CAR-T cells, bispecific antibodies, and T-cell engagers, actively recruit cytotoxic immune cells into otherwise poorly infiltrated tumors and therefore offer a fundamentally different strategy from conventional immune checkpoint blockade. Nevertheless, durable clinical benefit remains constrained by persistent stromal barriers, CAF-mediated immune suppression, myeloid-driven immunosuppressive signaling, and limited immune-cell persistence [9,112,114]. These findings indicate that successful immunotherapy in PDAC requires not only effective immune-cell recruitment but also restoration of a permissive tumor microenvironment capable of sustaining antitumor immunity.

Collectively, these findings support a therapeutic framework in which each treatment modality fulfills a distinct but complementary biological function. KRAS-targeted therapies suppress the primary oncogenic driver, DDR-directed therapies exploit treatment-induced vulnerabilities associated with replication stress and genomic instability, and immune-redirection strategies provide the effector mechanism for durable tumor elimination once immune access has been restored. Rather than representing competing therapeutic options, these approaches should therefore be considered complementary components of an integrated treatment strategy designed to overcome the interconnected adaptive resistance network that characterizes PDAC.

Future clinical progress will depend not only on the development of more effective individual agents but also on identifying the optimal biological context, treatment sequence, and biomarker-guided patient selection for each therapeutic modality. Longitudinal molecular monitoring, adaptive biomarkers, and rationally designed combination trials will be essential to determine when and how these complementary approaches should be integrated into clinical practice. Ultimately, translating this biologically informed framework into prospective biomarker-driven studies may provide a more durable and personalized therapeutic strategy for patients with PDAC.

## 8. Outstanding Research Priorities

Despite recent therapeutic advances, several unresolved questions will determine whether emerging strategies produce durable improvements in PDAC outcomes.

First, biomarker development must move beyond static genomic classification. KRAS mutation subtype and germline BRCA status remain important selection tools, but they do not adequately capture functional replication stress, treatment-induced homologous recombination deficiency, stromal composition, immune competence, or antigen heterogeneity. Prospective studies should therefore incorporate functional DDR assays, transcriptional and proteomic signatures, spatial immune profiling, and longitudinal assessment of surface targets such as CLDN18.2.

Second, mechanisms of resistance should be monitored during rather than only after treatment. Serial circulating tumor DNA analysis may enable early detection of secondary KRAS alterations, pathway reactivation, and emerging resistant clones. Paired tumor biopsies, when feasible, could further characterize changes in lineage state, YAP/TAZ activity, stromal architecture, and immune-cell composition. Such longitudinal analyses are particularly important for therapies vulnerable to antigen loss or treatment-induced phenotypic plasticity.

Third, the optimal sequence of KRAS-targeted, DDR-directed, and immune-redirection therapies remains undefined. Prospective studies should determine whether DDR vulnerabilities are best exploited concurrently with KRAS inhibition or after the emergence of treatment-induced replication stress. Similarly, immune-redirection platforms may require prior or concurrent stromal and myeloid remodeling to achieve adequate trafficking and effector-cell persistence.

Fourth, future combination trials should be based on experimentally validated resistance mechanisms rather than empirical addition of active agents. Adaptive platform trials incorporating biomarker-defined cohorts, predefined resistance hypotheses, serial molecular monitoring, and early stopping rules may provide an efficient framework for evaluating multidimensional treatment strategies. Mandatory translational endpoints should include pharmacodynamic target engagement, clonal evolution, immune trafficking, and stromal remodeling.

Finally, negative clinical results should be used to refine biological selection rather than simply abandon therapeutic targets. The failures of PEGPH20, non-personalized cancer vaccines, and unselected autophagy inhibition illustrate that strong preclinical rationale does not ensure clinical benefit in biologically heterogeneous PDAC. Identifying the disease stage, microenvironmental context, and functional biomarkers associated with therapeutic vulnerability will therefore be essential for future development.

Addressing these priorities may allow PDAC treatment to evolve from static mutation-matched therapy toward dynamic, resistance-informed precision oncology.

## 9. Conclusions

Recent developments have established that several major biological drivers of PDAC, including oncogenic KRAS signaling, DDR dependency, tumor-associated surface antigens, and stromal immune exclusion, are therapeutically actionable. The phase III success of daraxonrasib provides important clinical validation of direct RAS targeting, while emerging DDR-directed and immune-redirection strategies are expanding the range of potentially treatable biological subsets. Nevertheless, early responses remain vulnerable to pathway reactivation, phenotypic plasticity, metabolic adaptation, antigen heterogeneity, and stromal-mediated immune suppression.

Durable clinical progress is therefore unlikely to result from uniform application of a single therapeutic class. Future strategies should integrate baseline molecular selection with longitudinal monitoring of treatment-induced biological states and resistance mechanisms. Biomarker-guided sequencing, mechanism-based combinations, and adaptive clinical trial designs will be essential to translate recent therapeutic advances into sustained improvements in outcomes for patients with PDAC.

## Figures and Tables

**Table 1:** Current Standard-of-Care Regimens in Advanced PDAC.

Regimen	Clinical Setting	Trial (N)	ORR	mPFS	mOS	Major Toxicities	Ref.
FOLFIRINOX	1L metastatic PDAC	PRODIGE 4 (N = 342)	31.6%	6.4 mo	11.1 mo	Neutropenia, diarrhea, neuropathy	[5]
Gemcitabine + nab-paclitaxel	1L metastatic PDAC	MPACT (N = 861)	23%	5.5 mo	8.5 mo	Neuropathy, neutropenia	[6]
NALIRIFOX	1L metastatic PDAC	NAPOLI-3 (N = 770)	41.8%	7.4 mo	11.1 mo	Diarrhea, nausea, neutropenia	[20]

ORR, objective response rate; mPFS, median progression-free survival; mOS, median overall survival; PDAC, pancreatic ductal adenocarcinoma. Major toxicities refer to treatment-related adverse events of grade ≥3 according to the Common Terminology Criteria for Adverse Events (CTCAE), where reported.

## Data Availability

No new data were created or analyzed in this study. Data sharing is not applicable to this article.

## Abbreviations

The following abbreviations are used in this manuscript:

ADC	Antibody-drug conjugate
ATR	ataxia telangiectasia and Rad3-related
APC	Antigen-presenting cell
CAF	Cancer-associated fibroblast
CAR-T	Chimeric antigen receptor T-cell
CDK	Cyclin-dependent kinase
CEA	Carcinoembryonic antigen
CLDN18.2	Claudin18.2
CSF1R	Colony-stimulating factor 1 receptor
CTLA-4	Cytotoxic T-lymphocyte-associated protein 4
DCR	Disease control rate
DDR	DNA damage response
EMT	Epithelial–mesenchymal transition
FAK	Focal adhesion kinase
FDA	Food and Drug Administration
GEJ	Gastroesophageal junction
GnP	Gemcitabine plus nab-paclitaxel
HRD	Homologous recombination deficiency
ICI	Immune checkpoint inhibitor
IL	Interleukin
KRASi	KRAS inhibitor
MAPK	Mitogen-activated protein kinase
MDSC	Myeloid-derived suppressor cell
mFOLFIRINOX	Modified fluorouracil, leucovorin, irinotecan, and oxaliplatin
MSI-H	Microsatellite instability-high
mOS	Median overall survival
mPFS	Median progression-free survival
mTOR	Mammalian target of rapamycin
ORR	Objective response rate
OS	Overall survival
PARP	Poly(ADP-ribose) polymerase
PD-1	Programmed cell death protein 1
PDAC	Pancreatic ductal adenocarcinoma
PDO	Patient-derived organoid
PD-L1	Programmed death-ligand 1
PI3K	Phosphoinositide 3-kinase
PRMT5	Protein arginine methyltransferase 5
RAS(ON)	Active GTP-bound RAS state
RTK	Receptor tyrosine kinase
SHP2	Src homology region 2-containing protein tyrosine phosphatase 2
STING	Stimulator of interferon genes
TAM	Tumor-associated macrophage
TCE	T-cell engager
TGF-β	Transforming growth factor-beta
TME	Tumor microenvironment
TNKS	Tankyrase
Treg	Regulatory T cell
WEE1	WEE1 G2 checkpoint kinase
YAP/TAZ	Yes-associated protein/transcriptional coactivator with PDZ-binding motif
